# Enhanced nitrate removal in aquatic systems using biochar immobilized with algicidal *Bacillus* sp. AK3 and denitrifying *Alcaligenes* sp. M3: A synergistic approach

**DOI:** 10.1371/journal.pone.0318416

**Published:** 2025-03-05

**Authors:** Khomsan Ruangrit, Kittiya Phinyo, Sahassawat Chailungka, Kritsana Duangjan, Apitchaya Naree, Jearanai Thasana, Wassana Kamopas, Senoch Seanpong, Jeeraporn Pekkoh, Nuttapol Noirungsee

**Affiliations:** 1 Multidisciplinary Research Institute, Chiang Mai University, Chiang Mai, Thailand; 2 Office of Research Administration, Chiang Mai University, Chiang Mai, Thailand; 3 Department of Biology, Faculty of Science, Chiang Mai University, Chiang Mai, Thailand; 4 Thermal System Research Unit, Multidisciplinary Research Institute, Chiang Mai University, Chiang Mai, Thailand; 5 Department of Biology, Institute of Plant Science and Microbiology, University of Hamburg, Hamburg, Germany; 6 Environmental Science Research Center, Faculty of Science, Chiang Mai University, Chiang Mai, Thailand; 7 Research Center of Microbial Diversity and Sustainable Utilization, Faculty of Science, Chiang Mai University, Chiang Mai, Thailand; Gujarat Institute of Desert Ecology, INDIA

## Abstract

This study investigates the effectiveness of biochar immobilized with algicidal *Bacillus* sp. AK3 and denitrifying *Alcaligenes* sp. M3 in mitigating harmful algal blooms (HABs) and reducing nitrate pollution in aquatic environments. Over a six-day period, we analyzed changes in algal bloom-forming *Microcystis* density, chlorophyll-a levels (indicative of algal biomass), nitrate concentration, and microbial community composition in water treated with biochar and *Bacillus* sp. AK3 and *Alcaligenes* sp. M3-immobilized biochar. In water treatment using the AK3 and M3-immobilized biochar, *Microcystis* density decreased from 600,000 cells/mL to 80,000 cells/mL, and chlorophyll-a concentrations also substantially reduced, from 85.7 µg/L initially to 42.8 µg/L. Nitrate concentrations in the AK3 and M3-immobilized biochar treatment significantly decreased from approximately 23 mg/L to around 14 mg/L by Day 6, demonstrating the enhanced denitrification capabilities of the immobilized *Alcaligenes* sp. M3 and associated bacterial communities. The results also showed significant shifts in bacterial communities, with a decrease in *Microcystis*, highlighting the specific algicidal activity of *Bacillus* sp. AK3. The study underscores the potential of biochar-based treatments as a sustainable and effective approach for improving water quality and mitigating the environmental impacts of nutrient pollution and HABs.

## Introduction

The proliferation of these harmful algal blooms (HABs) is often attributed to nutrient enrichment, particularly in freshwater ecosystems, where they can create unsightly dense mats. These unsightly mats disrupt aquatic life and recreational activities. Toxic algal blooms caused by microcystin-producing cyanobacteria pose significant environmental and public health challenges. Microcystins are hepatotoxins that can cause liver damage in humans and animals upon ingesting contaminated water [1]. Microcystins are primarily produced by species within the genus *Microcystis*, which thrive in nitrogen and phosphorus-rich conditions [2]. Among these nutrients, nitrate plays a crucial role in fueling the growth of these cyanobacteria. Elevated nitrate levels in water bodies often result from agricultural runoff, sewage discharge, and industrial effluents, leading to eutrophication. This process accelerates algal growth, including that of toxic cyanobacteria, thereby exacerbating the incidence and severity of toxic algal blooms [3]. These algal toxins are resilient to degradation and can persist in water bodies, leading to prolonged exposure risks [4,5]. Algicidal bacteria have demonstrated considerable promise in suppressing the growth of microcystin-producing cyanobacteria, providing an effective approach for addressing microcystins contamination in freshwater [6]. These bacteria can inhibit or lyse cyanobacterial cells, thereby reducing the biomass of toxic algae and the concentration of microcystins in the water through various mechanisms. By integrating algicidal bacteria into water management strategies, it is possible to achieve a biological means of controlling harmful algal blooms, which is a desirable alternative to chemical treatments that can have adverse environmental impacts [7,8].

Nitrate pollution can be effectively mitigated through the action of aerobic denitrifiers. These microorganisms convert nitrate into nitrogen gas through a process known as aerobic denitrification, thereby removing excess nitrogen from the water [9]. By reducing nitrate concentrations, aerobic denitrifiers can indirectly limit the nutrient availability for cyanobacterial growth, thus contributing to the control of HABs. This biological approach to nitrate removal aligns well with sustainable water management practices to reduce nutrient pollution and its downstream effects.

Biochar, a carbon-rich product derived from the pyrolysis of organic matter, has been shown to enhance the growth and performance of various microorganisms, including those with bioremediation capabilities [10]. Biochar’s porous structure and large surface area provide an ideal habitat for microbial colonization. This study seeks to immobilize two bacterial strains, *Bacillus* sp. AK3 and *Alcaligenes* sp. M3, onto biochar to simultaneously address the over-grown *Microcystis* and nitrate pollution in freshwater reservoirs. The *Bacillus* species was used due to its reported effectiveness against *Microcystis* and its ability to be immobilized in porous materials [7]. The *Alcaligenes* species was isolated from a water treatment system utilizing biochar as a substrate. The combination of these two species was evaluated to investigate their potential synergistic effects in mitigating *Microcystis* growth and improving water quality. By leveraging the synergetic effects of biochar-supported microbial activity, this approach aims to develop an efficient and sustainable method for mitigating harmful algal blooms and nutrient pollution in aquatic ecosystems.

## Methods

### Biochar preparation

The biochar produced from bamboo biomass was provided by the Faculty of Engineering, Chiang Mai University, Thailand. Briefly, the biochar production process involved drying the biomass at 80°C for 24 hours. Following the drying process, each dried biomass sample was subjected to a slow pyrolysis at 500°C for 2 hours in a fixed-bed reactor [11].

### Immobilization of bacteria on biochar

The characteristics and algicidal activity of *Bacillus* sp. AK3 were described in a previous study [7]. *Alcaligenes* sp. M3 was previously isolated from a constructed wetland. Stock cultures of the two bacterial species were cultivated in Luria Bertani broth (LB broth) medium with shaking at 20 rpm at room temperature (25.0 ± 2.00 °C) for 48 hours. The initial cell density of *Bacillus* sp. AK3 and *Alcaligenes* sp. M3 were OD600 =  0.100 and 0.800, respectively. Then were inoculated into the 4 liters of sterile LB broth in a 10-liter Nalgene™ Round Polycarbonate Clearboy™ Carboy (Thermo Fisher Scientific Inc.) at a total inoculum volume of 10% (v/v) and incubated for 48 hours at room temperature. Then 200 g of biochar was added, and the incubation continued for another 48 hours. The *Bacillus* sp. AK3 and *Alcaligenes* sp. M3-immobilized biochar would then be ready for use in further experiments.

### Experimental design

The experiment was conducted in 10-liter column tanks. The setup consisted of 8 liters of natural water collected from Angkaew Reservoir, Chiang Mai University, Chiang Mai, Thailand (18°48’22.2“N 98°57’03.2”E) and 2 liters of stationary phase *Microcystis* sp. AARL C028 cultured in BG-11 medium. The mixture was adjusted to achieve an initial cell density of 600,000 cells/ml for *Microcystis*. The experiment included six replicates under three conditions: control (10 liters of the water sample described above), biochar control (200 g of biochar in 10 liters of the water sample described above), and biochar charged with bacteria (200 g of biochar with bacteria in 10 liters of the water sample described above).

The experiment was conducted for 6 days. The water parameters, including water temperature, pH, and conductivity, were measured by a multimeter (PONPE 510PD, Photonics). The growth of algae was monitored by measuring chlorophyll using a spectrophotometer at the wavelengths of 560 nm, 680 nm, and 750 nm. The *Microcystis* cell density was measured through direct counting, following the method previously described [7]. Two samples were taken for each replicate, and the cell count per milliliter was determined. The counts from these two samples were averaged to obtain a representative value for that replicate. Finally, the cell densities from all six replicates were averaged to determine the overall cell density for the treatment. Nutrients including ammonium-nitrogen (NH_4_^ + ^-N), nitrate-nitrogen (NO_3_^—^N), and orthophosphate (PO_4_^3-^) were determined by the Nesslerization method, cadmium reduction method, and ascorbic acid method, respectively, using Hach DR/2010 spectrophotometer (Hach Company). These measurements were performed on Days 0, 3, and 6 of the experiment.

### Amplicon metagenomic sequencing analysis

Water samples of 30 ml were collected on Days 0, 3, and 6 for DNA extraction using the DNeasy PowerWater Kit (Qiagen) according to the manufacturer’s protocols. Microbial communities were analyzed using QIIME2 (version 2023.7) [12]. The primers and sequences arising from sequencing error were removed for the bacterial community analysis with Cutadapt [13]. Subsequently, the chimera reads were filtered, and the amplicon sequence variants (ASVs) with DADA2 were identified [14]. Alpha rarefaction maintained a consistent slope relative to the sequencing depth, indicating that the sequencing depth within each sample was adequate for capturing the diversity of microbial communities. Sequences from the Silva 138 database underwent the extraction with a set of primers 338F 3’-ACTCCTACGGGAGGCAGCA-5’ and 806R 3’-GGACTACHVGGGTWTCTAAT-5’ to generate reference sequences that specifically cover V3-V4 region, and then classifier was trained using reference sequences [15,16]. Afterward, the bacterial classification was classified with the trained classifier. The mitochondria, chloroplast, and archaea were filtered using the R package phyloseq (version 1.42.0) [17]. Data are available at The European Nucleotide Archive (ENA) under accession number PRJEB79565.

### Statistical analyses

Statistical analyses were conducted to evaluate the effects of treatments and time (days) on various environmental parameters, including nitrate, phosphorus, ammonium, pH, conductivity, Biological Oxygen Demand (BOD), chlorophyll concentration, and *Microcystis* cell count. A two-way analysis of variance (ANOVA) was performed for each parameter to assess the main effects of treatment, day, and their interaction. Tukey’s Honest Significant Difference (HSD) post-hoc test was applied to identify significant differences between treatments within each day. All statistical analyses were performed in R, utilizing the aov function for ANOVA and the TukeyHSD function for post-hoc tests. Graphical representations were created using the ggplot2 package [18]. Statistical significance was determined at a threshold of p <  0.05.

To ensure impartial microbial diversity analysis, rarefy was performed by selecting a uniform number at sequencing depth that fully captured all samples. Subsequently, the bacterial relative abundance was illustrated in a stacked plot using R package ggplot2 (version 3.4.4) [18] in R studio [19]. Shannon diversity and Observed features were performed using R package phyloseq (version 1.42.0) to estimate the alpha diversity of microbial communities in treatments. Wilcoxon’s rank sum test was used to assess the statistical significance of the difference between treatments. The analysis was further visualized using the R package ggplot2 (version 3.4.4). Beta diversity analysis and the Principal Coordinates Analysis (PCoA) were conducted by using R package phyloseq to assess the dissimilarity of microbial communities across groups by using the Bray-Curtis distance. The differences among treatments in the microbial community were tested by the dissimilarities by permutational multivariate analysis of variance (PERMANOVA) using the adonis2 function in the vegan package (version 2.6-4) [20]. Functional prediction was performed with FAPROTAX [21] in the R package microeco (version 1.9.1) [22].

## Results

### Impact of biochar treatments on pH, conductivity, and biological oxygen demand

**Fig 1:**
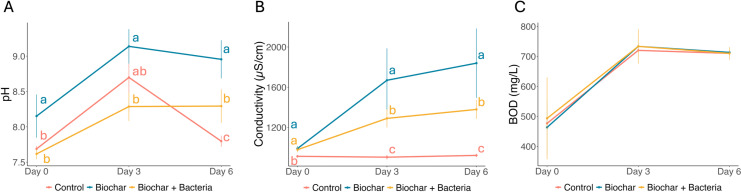
Effect of treatments on pH, conductivity, and biological oxygen demand (BOD) over time. (A) pH levels in Control, Biochar, and Biochar+Bacteria treatments from Day 0 to Day 6. The Biochar treatment shows the highest increase in pH, particularly by Day 3, with a subsequent decrease by Day 6. (B) Conductivity (µS/cm) across the same treatments over time. Conductivity increased significantly in the Biochar treatment, with the Biochar+Bacteria treatment also showing a notable rise, although to a lesser extent. (C) Biological Oxygen Demand (BOD) (mg/L) measured across treatments, indicating an increase by Day 3, followed by a slight moderation by Day 6. Error bars represent standard deviations from the mean. Letters above the data points represent significant differences between treatments on the same day (Tukey’s HSD test, p <  0.05). Comparisons are made only among treatments within each day and not across different days for the same treatment.

The Biochar treatment significantly increased pH levels compared to the Control, with the most pronounced increase observed on Day 3 (mean pH =  9.14, Fig 1A). The Biochar+Bacteria treatment also elevated pH to a lesser extent (mean pH =  8.29 by Day 3). Two-way ANOVA confirmed significant effects of treatment and time on pH (both p <  0.001), with a significant interaction (p <  0.001). Tukey’s HSD test revealed that the Biochar treatment had the strongest impact on pH, significantly differing from the Control on Days 3 and 6. These findings indicate that Biochar, alone or with bacteria (AK3 and M3), influences water pH, potentially affecting water quality management.

Conductivity was also higher in the Biochar and Biochar+Bacteria treatments compared to the Control, particularly on Day 3 (mean values: 1668.83 µ S/cm and 1289.50 µ S/cm, respectively, Fig 1B). Two-way ANOVA indicated significant effects of treatment, time, and their interaction (all p <  0.001). Post-hoc analysis showed that the Biochar+Bacteria treatment significantly increased conductivity compared to the Control on Days 3 and 6 (p <  0.01).

Biological Oxygen Demand (BOD) levels were not significantly affected by treatment, as two-way ANOVA indicated no significant effect of treatment (p =  0.864) or its interaction with time (p =  0.957, Fig 1C). While time alone had a significant influence (p <  0.001), these results suggest that temporal variations rather than experimental treatments drive BOD changes.

### Comparative effectiveness of biochar-based treatments on *Microcystis* density and chlorophyll levels

**Fig 2:**
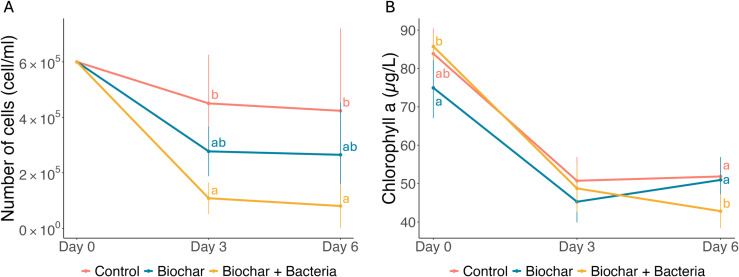
Effects of Biochar and Biochar+Bacteria treatments on *Microcystis* cell density and chlorophyll-a concentration over time. (A) Changes in *Microcystis* cell density (cells/mL) in Control, Biochar, and Biochar+Bacteria treatments from Day 0 to Day 6. The Biochar+Bacteria treatment shows a significant reduction in cell density, particularly by Day 6. (B) Chlorophyll-a concentrations (µg/L) in the same treatments over the same period, indicating the algal biomass. The Biochar+Bacteria treatment shows a pronounced reduction in chlorophyll-a levels, mirroring the reduction in *Microcystis* cell density. Error bars represent standard deviations from the mean. Letters above the data points represent significant differences between treatments on the same day (Tukey’s HSD test, p <  0.05). Comparisons are made only among treatments within each day and not across different days for the same treatment.

The Biochar+Bacteria treatment significantly reduced *Microcystis* density over six days, decreasing from 600,000 cells/mL on Day 0 to 80,333 cells/mL by Day 6 (p <  0.05, Fig 2A). In contrast, the Biochar treatment alone resulted in a smaller reduction, stabilizing at approximately 265,000 cells/mL by Day 6. The Control treatment showed a gradual decline to 423,667 cells/mL by Day 6. Two-way ANOVA indicated significant effects of treatment and time on cell density (p <  0.001), with a significant interaction (p =  0.026).

Similarly, chlorophyll levels mirrored *Microcystis* density trends. The Biochar+Bacteria treatment resulted in the greatest reduction in chlorophyll by Day 6 (mean =  43 µg/L), compared to 51 µg/L in the Control and Biochar treatments (Fig 2B). Two-way ANOVA showed significant effects of treatment and time (p <  0.05 and p <  0.001, respectively), with a significant interaction (p =  0.006). These findings demonstrate that combining bacteria (AK3 and M3) with Biochar enhances its effectiveness in reducing both *Microcystis* cell density and chlorophyll levels.

### The effects of treatments on nitrate, ammonium, and phosphorus levels in water

**Fig 3:**
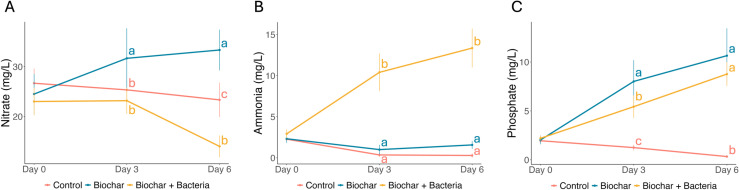
Impact of Biochar and Biochar+Bacteria treatments on nitrate, ammonia, and phosphate levels over time. (A) Nitrate concentration (mg/L) across Control, Biochar, and Biochar+Bacteria treatments from Day 0 to Day 6. The Biochar+Bacteria treatment shows a significant reduction in nitrate levels by Day 6, indicating effective denitrification. (B) Ammonia concentration (mg/L) in the same treatments, showing a marked increase in the Biochar+Bacteria treatment, which may suggest ammonia production through processes such as DNRA (Dissimilatory Nitrate Reduction to Ammonia). (C) Phosphate concentration (mg/L) over time, where both Biochar and Biochar+Bacteria treatments exhibit changes in phosphate levels, with a notable increase in the Biochar treatment. Error bars represent standard deviations from the mean. Letters above the data points represent significant differences between treatments on the same day (Tukey’s HSD test, p <  0.05). Comparisons are made only among treatments within each day and not across different days for the same treatment.

The Biochar+Bacteria treatment significantly reduced nitrate levels by Day 6 (14.0 mg/L), compared to 23.33 mg/L in the Control and 33.33 mg/L in the Biochar treatment (Fig 3A). Two-way ANOVA confirmed significant effects of treatment (p <  0.001), time (p =  0.034), and their interaction (p <  0.001). Tukey’s HSD test highlighted the superior performance of the Biochar+Bacteria treatment in nitrate reduction.

Ammonium levels increased in all treatments, with the most substantial rise observed in the Biochar+Bacteria treatment (from 2.92 mg/L on Day 0 to 13.35 mg/L on Day 6, Fig 3B). Two-way ANOVA revealed significant effects of treatment and time (both p <  0.001), suggesting that the combined treatment may enhance ammonium release.

Phosphorus levels were significantly elevated in both the Biochar and Biochar+Bacteria treatments compared to the Control (Fig 3C). However, no significant difference was observed between the two Biochar treatments. These results suggest that while Biochar increases phosphorus levels, the addition of AK3 and M3 does not exacerbate this effect.

### The effects of biochar on bacterial communities

**Fig 4:**
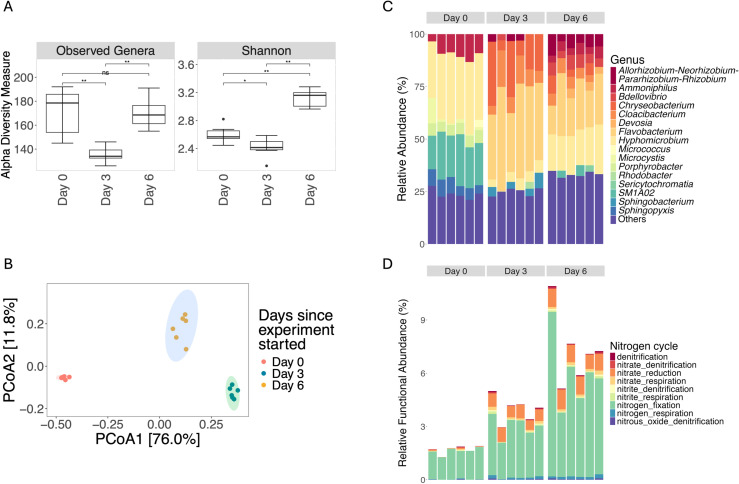
Changes in bacterial community diversity, composition, and function over time in Biochar+Bacteria treatment. (A) Alpha diversity measures of bacterial communities over time, as represented by observed genera and Shannon diversity indices. Both metrics show an initial drop on Day 3, followed by a rebound on Day 6. Statistical significance (Wilcoxon’s rank sum test) is indicated by *p* values: ** p <  0.01, *  p <  0.05, ns =  not significant. (B) Principal Coordinates Analysis (PCoA) plot based on Bray-Curtis dissimilarity, illustrating the separation of bacterial communities by day. The distinct clustering indicates significant changes in community structure over the course of the experiment. The ovals represent 95% confidence ellipses around the group centroids. (C) Relative abundance of bacterial genera over time, highlighting shifts in community composition. Notable changes in the relative abundance of key genera, including *Microcystis*, *Sphingopyxis*, and others, are observed. (D) Predicted functional profiles related to the nitrogen cycle, showing an increase in denitrification and other nitrogen-related functions by Days 3 and 6, consistent with changes in microbial composition and activity.

The application of bacteria-immobilized biochar altered the diversity of bacterial communities. The richness and Shannon diversity at the genus level initially dropped on Day 3 but rebounded by Day 6 (Fig 4A). Beta diversity analysis, based on the Bray-Curtis dissimilarity matrix and PCoA, showed distinct groupings (Fig 4B). PERMANOVA indicated that the communities on different days were significantly different (p <  0.05). This suggests that the application of charged biochar led to changes in bacterial community structures over time.

On Day 0, the initial bacterial community composition shows a higher relative abundance of genera such as *Ammoniphilus*, *Hyphomicrobium*, *Microcystis*, *Porphyrobacter*, SM1A02, and *Sphingopyxis* (Fig 4C). This initial composition provides a baseline for understanding how the bacterial communities change over time with the treatment. By Day 3, there is a noticeable shift in the bacterial community structure. *Chryseobacterium*, *Cloacibacterium* and *Flavobacterium* became dominant, indicating a positive response to the treatment. Genera like *Ammoniphilus*, *Hyphomicrobium*, *Microcystis*, *Porphyrobacter*, SM1A02 and *Sphingopyxis* decrease compared to Day 0, suggesting that the treatment may be suppressing these bacteria. By day 6, the bacterial community structure shifts further, with *Hyphomicrobium* and *Ammoniphilus* rebound to become more dominant compared to Day 3. Along with other dominant genera such as *Allorhizobium-Neorhizobium-Pararhizobium-Rhizobium*, *Bdellovibrio*, and *Flavobacterium*. *Chryseobacterium* and *Cloacibacterium* was present but in varying proportions compared to earlier days. *Microcystis* was present at low abundance in all groups (S1 Fig). This result confirmed the algal density experiment, which showed that *Microcystis* decreased in density when treated with charged biochar. Functional prediction showed increasing trends in denitrification functions (Fig 4D), along with a decreasing trend in photosynthesis (S2 Fig). These shifts suggest that the application of biochar-immobilized bacteria may effectively alter microbial communities, potentially aiding in the mitigation of *Microcystis* and the removal of nitrate.

## Discussion

The rationale for combining *Bacillus* sp. AK3 and *Alcaligenes* sp. M3 lies in their complementary functional roles and the potential for synergistic effects. *Bacillus* sp. AK3 is an algicidal bacterium that specifically targets and reduces *Microcystis* populations, thereby controlling harmful algal blooms. Meanwhile, *Alcaligenes* sp. M3, a denitrifying bacterium, contributes to nitrate reduction and improves water quality by mitigating nutrient pollution. Immobilizing these two species on biochar allows for a dual-action approach: *Bacillus* sp. AK3 effectively controls algal blooms, while *Alcaligenes* sp. M3 enhances denitrification, addressing both the ecological and nutrient-related factors of aquatic systems. This combined treatment was investigated to explore its potential synergistic benefits in achieving sustainable water quality management.

In our experimental setup, which included Control, Biochar, and Biochar+Bacteria treatments, *Microcystis* cells decreased over time regardless of the treatment. This decline may be due to non-optimal conditions in the setup. *Microcystis* are known to thrive in nutrient-rich environments [2,23,24]. However, during the experiment, 2 liters of BG11 containing the cells were added to 8 liters water from the reservoir. This abrupt change in environment may have impacted the establishment of *Microcystis* [25], resulting in reduced fitness and a decline in the population across all treatments. Albeit the non-optimal conditions for *Microcystis*, this mesocosm experiment was designed to closely resemble conditions of Angkeaw Reservoir, where harmful algal blooms occur.

However, the Biochar+Bacteria treatment resulted in a significantly lower cell count, particularly by Day 6. This was confirmed by an amplicon metagenomic study, which showed that the relative abundance of *Microcystis* decreased over time. *Bacillus* sp. AK3 has been shown to lyse *Microcystis* cells effectively and degrade microcystin [7,8]. Biochar has been shown to support various microorganisms in addressing environmental challenges, such as soil conditioning and water quality improvement, due to its porous nature, which provides an ideal habitat for microorganisms [26]. This is the first study to immobilize *Bacillus* sp. AK3 on biochar and investigate its algicidal activity. Our results demonstrate that *Bacillus* sp. AK3 remains active in killing *Microcystis* cells. However, this study did not include an analysis of microcystin levels. Further research is needed to elucidate the effectiveness of microcystin hydrolysis by *Bacillus* sp. AK3 immobilized on biochar.

One argument could be that the algae were not killed by the algicidal activity of *Bacillus* sp. AK3, but were instead affected by changes in water parameters resulting from the application of biochar. Biochar application alters various water parameters, including conductivity, pH, phosphate, nitrate, and ammonia. The conductivity, pH, and phosphate are dependent of feedstock and pyrolysis temperature of the biochar [27]. While biochar can absorb nutrients from the water, it can also release its contents into the environment [28]. Interestingly, when charged biochar was used, these increases in conductivity and pH, but not phosphate, were mitigated, possibly due to biofilm formation on the biochar that prevents the diffusion of its components into the water [29]. This highlights the advantage of using biochar charged with microorganisms to mitigate the potential negative impacts of biochar application.

*Microcystis* has been shown to thrive optimally within a pH range of 7 to 9 [30], so the increase in pH is unlikely to be the cause of its decline. The threshold electrical conductivity for *Microcystis* growth is 7.5 mS/cm [31], which is well above the highest conductivity observed in our study (2.3 mS/cm). Phosphorus generally promotes the growth of *Microcystis* [32]. Morever, the orthophosphate concentration in BG11 is approximately 21.85 mg/L, but the maximum orthophosphate level reached in the experiment was 15.4 mg/L, which is well below the cultured conditions. Therefore, phosphate toxicity is also unlikely to be the cause of *Microcystis* death. The cause of the reduction in *Microcystis* cells in the Biochar-alone treatment remains unclear, but it may result from unknown compounds released by the biochar. Therefore, a detailed study on the toxicity of biochar to *Microcystis* is warranted.

Nitrate has been shown to promote the growth of *Microcystis,* while high ammonia concentrations (>7 mg/L) have been shown to suppress *Microcystis* growth [33]. In our study, the nitrate concentration in the Biochar+Bacteria treatment was 14 mg/L, which is within the range that promotes *Microcystis* growth [34]. However, the ammonia concentration was 13 mg/L, which could potentially suppress *Microcystis* growth [33,34]. The effect of ammonia, as well as other nitrogen sources, on *Microcystis* can vary from strain to strain [35–37]. A detailed study is necessary to determine whether the increase in ammonia in the Biochar+Bacteria treatment is responsible for the decrease in *Microcystis* cell count.

Overall, the altered water parameters following the application of biochar are unlikely to be the cause of *Microcystis* cell death. Instead, *Bacillus* sp. AK3 immobilized on biochar is likely the effective algicidal agent.

In terms of nitrate removal, the Biochar+Bacteria treatment was effective in reducing nitrate levels in the water. *Alcaligenes* is known for its denitrification abilities and is commonly used in wastewater treatment to remove nitrate [38,39]. The reason *Alcaligenes* was not detected as one of the dominant genus in the microbial communities could be that it resides primarily within the biochar, rather than in the water, from which the samples were collected for metagenomic study. Biochar itself has been shown to promote denitrification by stimulating indigenous microorganisms [9]. The application of biochar has been reported to promote anammox and denitrifying bacteria, such as *Ca.* Brocadia, *Ca.* Kuenenia, *Pseudomonas*, *Thauera*, and *Dokdonella* [40]. In our experiment, we did not observe members of the *Brocadiaceae* family, which includes anammox bacteria. However, we did find known denitrifiers such as *Pseudomonas*, *Thauera*, *Paracoccus*, *Bacillus*, *Achromobacter*, and *Rhodobacter*. Functional prediction analysis also indicated an increase in denitrification activity on Days 3 and 6. These results suggest that the application of biochar charged with microorganisms orchestrates nitrate removal by utilizing both the biochar itself and the added microorganisms, while also reducing the potential negative effects of biochar such as phosphate leaching on water quality.

Interestingly, an increase in ammonia was observed only in the Biochar+Bacteria treatment. This could be due to several factors, such as decomposition or dissimilatory nitrate reduction to ammonia (DNRA). When *Bacillus* sp. AK3 lyses *Microcystis* cells, it results in an increase in organic matter similar to the degradation of *Microcystis* bloom in lake water [41]. Microorganisms can decompose this organic matter, releasing ammonia through ammonification, which aligns with our observation of increased chemoheterotrophic activity. However, this does not explain why this phenomenon was not observed in the Control and Biochar-alone treatments, as there was also an initial decrease in *Microcystis* count and chlorophyll content in those treatments. We speculate that the additional ammonia could result from DNRA. The ingrowth of *Clostridium*, a bacterium capable of performing DNRA [42], was observed in our study. The multispecies biofilm produced by *Bacillus* sp. AK3 and *Alcaligenes* sp. M3 could provide a suitable habitat for *Clostridium* to carry out DNRA, fueled by the available carbon from lysed *Microcystis* cells, thus converting nitrate to ammonia. This raises concerns regarding the application of charged biochar, as further optimization is needed to prevent such phenomena. The goal should be to ensure that the treatment solves the intended problem without inadvertently creating new issues.

Biochar has been suggested for water quality improvement, particularly its ability to support microbial growth and enhance biological processes [26]. They found that biochar could effectively host beneficial microbes, improving water quality. The current study corroborates these findings by demonstrating that biochar supports algicidal and denitrifying bacteria. This combined approach leads to significant improvements in water quality by simultaneously reducing algal biomass and nitrate concentrations. [5] provided guidelines for managing toxic cyanobacteria, including chemical treatments and physical barriers. While these methods can be effective in the short term, they often come with environmental drawbacks and high costs. In contrast, the current study demonstrates a biological method that not only mitigates algal blooms but also improves water quality by reducing nitrate levels. This method is potentially more sustainable and environmentally friendly than the traditional chemical and physical methods.

## Conclusion

This study is the first to use a synergistic approach to both reduce toxic algal blooms and mitigate nitrate pollution by loading algicidal *Bacillus* and aerobic denitrifiers onto biochar. This microbially-loaded biochar method reduced *Microcystis* cell counts and removed nitrate from the water. Additionally, the microorganisms helped minimize the release of unwanted biochar residues, such as phosphate, into the water. This demonstrates the promise of this approach in environmental management and water quality improvement. However, further research is needed to optimize the application of this charged biochar to prevent unwanted microbial activities, such as the increase in ammonia, before translating this approach into larger-scale applications.

## Supporting information

S1 Fig Relative abundance of *Microcystis* over time in Biochar+Bacteria treatment. The relative abundance of *Microcystis* (% of total bacterial community) was measured on Days 0, 3, and 6. A significant reduction in *Microcystis* abundance is observed over time, with the highest abundance recorded on Day 0, followed by a marked decline by Day 3 and minimal levels by Day 6. This trend demonstrates the effective algicidal activity of *Bacillus* sp. AK3 when immobilized on biochar.
(TIF)

S2 Fig Functional prediction of biochar immobilized with bacteria (AK3 and M3) over a six-day experiment. Boxplots represent the predicted functional profiles of microbial communities associated with biochar immobilized with *Bacillus* sp. AK3 and *Alcaligenes* sp. M3 across Days 0, 3, and 6. The analysis highlights the relative abundance of various microbial functions, including aerobic and anaerobic chemoheterotrophy, denitrification, nitrate reduction, methanotrophy, and nitrogen fixation, among others. Notable functional changes are observed over time, indicating shifts in the microbial community’s metabolic potential in response to the biochar treatment. Each boxplot shows the interquartile range (IQR), with the median indicated by the central line and outliers represented as individual points.
(TIF)

S1 File Water quality data for treatments. Measurements of nitrate, phosphorus, ammonium, pH, conductivity, temperature, BOD, chlorophyll, and *Microcystis* cell count for Control, Biochar, and Biochar+Bacteria treatments on days 0, 1, 3, and 6 with six replicates each.
(CSV)
