# Supplementary figures and images for: Enhanced nitrate removal in aquatic systems using biochar immobilized with algicidal Bacillus sp. AK3 and denitrifying Alcaligenes sp. M3: A synergistic approach
Source: PLoS One. 2025 Mar 5;20(3):e0318416. doi: 10.1371/journal.pone.0318416 (PMC11882090; doi:10.1371/journal.pone.0318416)

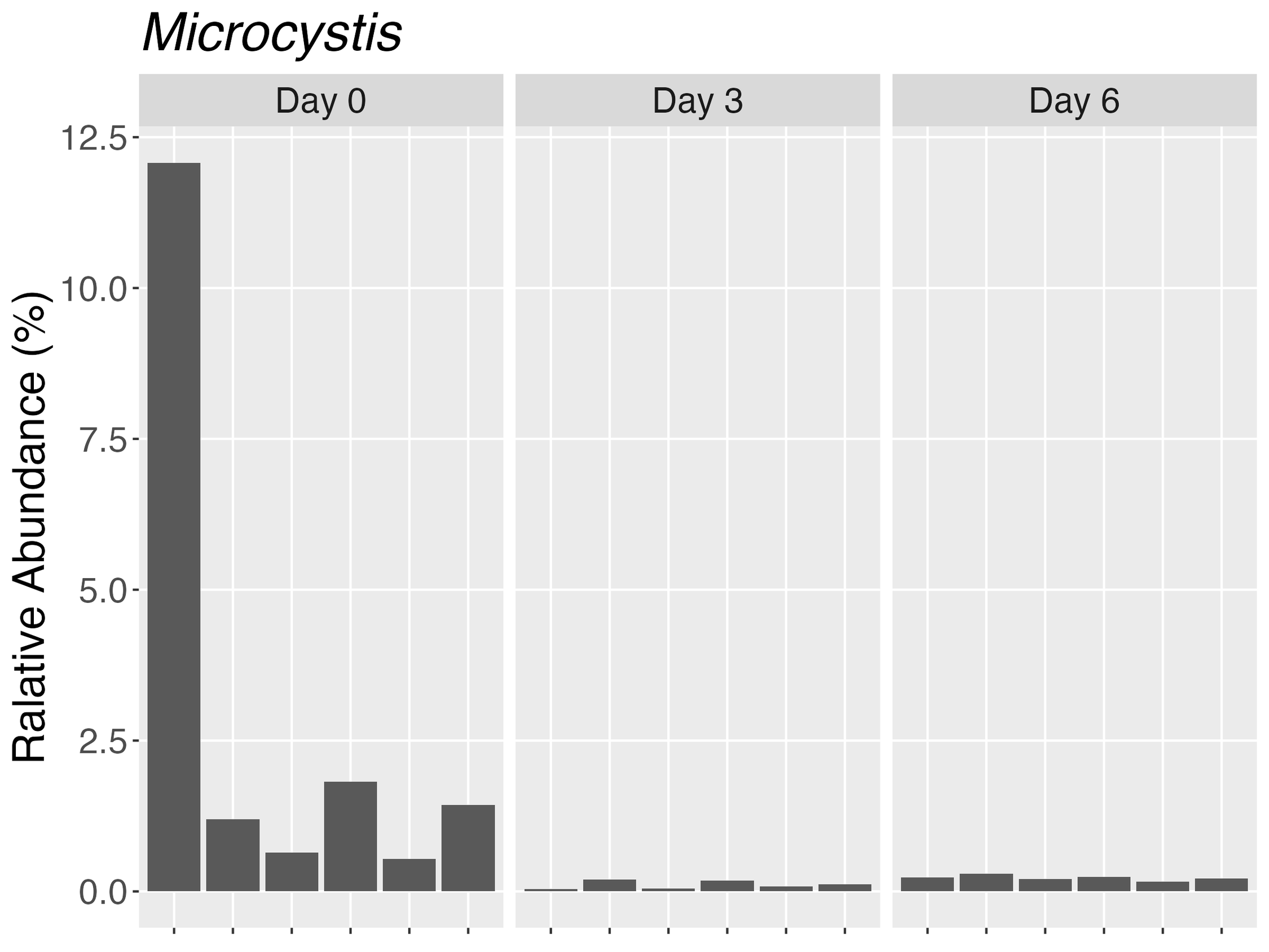

Supplement: S1 Fig — The relative abundance of Microcystis (% of total bacterial community) was measured on Days 0, 3, and 6. A significant reduction in Microcystis abundance is observed over time, with the highest abundance recorded on Day 0, followed by a marked decline by Day 3 and minimal levels by Day 6. This trend demonstrates the effective algicidal activity of Bacillus sp. AK3 when immobilized on biochar. (TIF) [file pone.0318416.s001.tif]

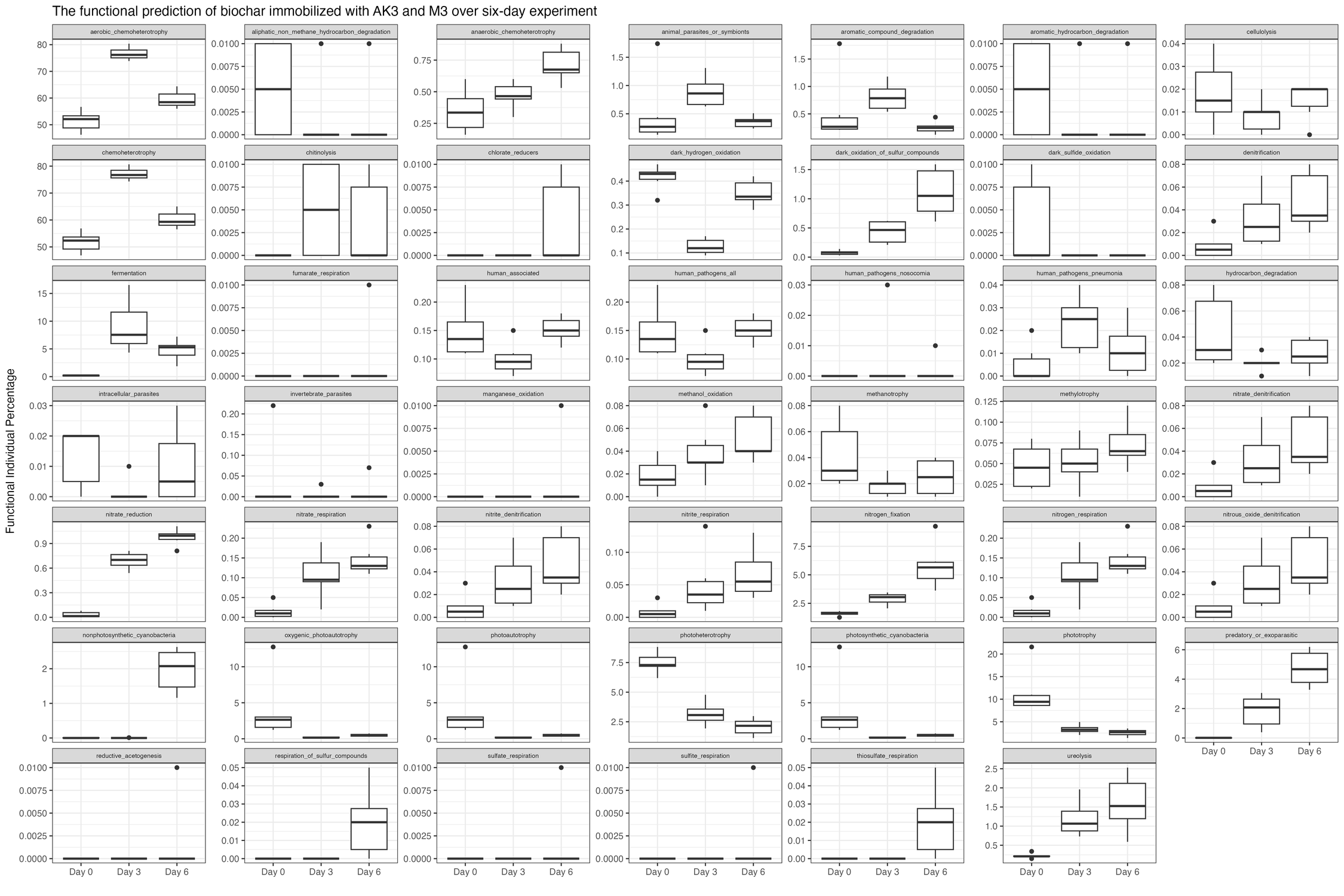

Supplement: S2 Fig — Boxplots represent the predicted functional profiles of microbial communities associated with biochar immobilized with Bacillus sp. AK3 and Alcaligenes sp. M3 across Days 0, 3, and 6. The analysis highlights the relative abundance of various microbial functions, including aerobic and anaerobic chemoheterotrophy, denitrification, nitrate reduction, methanotrophy, and nitrogen fixation, among others. Notable functional changes are observed over time, indicating shifts in the microbial community’s metabolic potential in response to the biochar treatment. Each boxplot shows the interquartile range (IQR), with the median indicated by the central line and outliers represented as individual points. (TIF) [file pone.0318416.s002.tif]
